# Determinants for use of direct-to-consumer telemedicine consultations in primary healthcare—a registry based total population study from Stockholm, Sweden

**DOI:** 10.1186/s12875-021-01481-1

**Published:** 2021-06-26

**Authors:** Cecilia Dahlgren, Margareta Dackehag, Per Wändell, Clas Rehnberg

**Affiliations:** 1 Department of Learning, Informatics, Management and Ethics, Karolinska Institutet, Stockholm, Sweden; 2 Region Stockholm, Center for Health Economics, Informatics and Healthcare Research, Stockholm, Sweden; 3 Department of Economics, Lund University, Lund, Sweden; 4 Department of Neurobiology, Care Sciences and Society, Division of Family Medicine and Primary Care, Karolinska Institutet, Stockholm, Sweden

**Keywords:** Telemedicine, eHealth, Primary healthcare, Healthcare utilization, Equity

## Abstract

**Background:**

In recent years, telemedicine consultations have evolved as a new form of providing primary healthcare. Telemedicine options can provide benefits to patients in terms of access, reduced travel time and no risk of disease spreading. However, concerns have been raised that access is not equally distributed in the population, which could lead to increased inequality in health. The aim of this paper is to explore the determinants for use of direct-to-consumer (DTC) telemedicine consultations in a setting where telemedicine is included in the publicly funded healthcare system.

**Methods:**

To investigate factors associated with the use of DTC telemedicine, a database was constructed by linking national and regional registries covering the entire population of Stockholm, Sweden (*N* = 2.3 million). Logistic regressions were applied to explore the determinants for utilization in 2018. As comparators, face-to-face physician consultations in primary healthcare were included in the study, as well as digi-physical physician consultations, i.e., telemedicine consultations offered by traditional primary healthcare providers also offering face-to-face visits, and telephone consultations by nurses.

**Results:**

The determinants for use of DTC telemedicine differed substantially from face-to-face visits but also to some extent from the other telemedicine options. For the DTC telemedicine consultations, the factors associated with higher probability of utilization were younger age, higher educational attainment, higher income and being born in Sweden. In contrast, the main determinants for use of face-to-face visits were higher age, lower educational background and being born outside of Sweden.

**Conclusion:**

The use of DTC telemedicine is determined by factors that are generally not associated with greater healthcare need and the distribution raises some concerns about the equity implications. Policy makers aiming to increase the level of telemedicine consultations in healthcare should consider measures to promote access for elderly and individuals born outside of Sweden to ensure that all groups have access to healthcare services according to their needs.

**Supplementary Information:**

The online version contains supplementary material available at 10.1186/s12875-021-01481-1.

## Background

Telemedicine consultations, where healthcare personnel and patients are spatially separated and interact through video-link, telephone or electronic chat, have emerged as a new mode of providing primary healthcare in Sweden and internationally in recent years. The use of telemedicine has increased substantially following the COVID-19 pandemic [1] and telemedicine will in all probability constitute a growing part of the health care sector in the future. The Swedish government and the Swedish Association of Local Authorities and Regions have endorsed a common vision for eHealth stating that Sweden will be best in the world at using the opportunities offered by digitization and eHealth by 2025 [2].

The introduction of telemedicine in the Swedish healthcare system has been dominated by actors operating in parallel with traditional primary care. These direct-to-consumers (DTC) telemedicine providers have often acted as subcontractors to a primary healthcare centre in one region while offering their services directly to patients nationally. Unlike common practice for healthcare providers in Sweden, some of the DTC providers have used broad advertising campaigns as a method to attract customers [3] and patients can access their services through mobile apps. Through an agreement between Swedish regions, the DTC telemedicine consultations are reimbursed by a fee-for-service principle and the services are a part of the publicly funded system. Following the popularity of the DTC telemedicine options, traditional primary healthcare providers have also developed telemedicine alternatives, conceptualized as digi-physical healthcare.

There are several potential benefits associated with telemedicine but also possible drawbacks. Telemedicine consultations have a potential to be cost saving for patients, providers and taxpayers. Moreover, there are benefits related to accessibility and reduced risk of spreading disease. However, concerns have also been raised that digital healthcare providers will increase the use of unnecessary care and transfer resources from those with higher needs to those with lower needs [4–6].

Results from studies from both Swedish and international settings indicate that users of telemedicine are younger and female to a further extent than the population as a whole or in comparison to users of face-to-face consultations in primary healthcare [7–13]. However, there are contrasting results regarding the impact of socioeconomic factors. Park and co-authors [14] found that telemedicine was used less by Medicaid beneficiaries and low income populations while Mehrotra and co-authors [8] found that telemedicine use among rural Medicare beneficiaries was associated with living in a poorer community. Other studies from English and US settings have found no association between the use of telemedicine and higher income or other patient socioeconomic factors [10, 15]. From the Swedish setting, Ekman and co-authors observed a positive association between telemedicine use and income at the municipality level [16]. Similarly, Ellegård and Kjellsson found a higher proportion of high income earners among telemedicine users in comparison to non-users [17].

Telemedicine offers reduced travel times and travel costs for patients [18], an advantage which indicates that the health service would be especially beneficial for those individuals who live at a far distance from their healthcare provider. However, also regarding distance to provider there are contrasting results. In a US setting, Mehrotra and co-authors [10] found that patients who sought care via digital providers in comparison to the control group who made traditional office visits had longer travel distance from patient home to clinic while Swedish findings indicate that the use is more widespread in the metropolitan regions than in the rest of the country [11, 16]. Similarly, Park and co-authors found that US citizens in rural areas were less likely to have used telemedicine [14].

The use of telemedicine will most likely expand in the future and it is important to monitor the use in order to ensure appropriate level of consumption and care on equal terms for the entire population. In this study we add to the knowledge base regarding determinants for the use of telemedicine and contribute with findings based on a large population dataset with individual level data on socioeconomic factors, healthcare history as well as factors regarding chosen primary healthcare provider such as distance to provider.

## Methods

### Aim

The aim of this study is to explore the determinants for use of DTC telemedicine consultations in a setting where telemedicine is included in the publicly funded healthcare system.

### Setting

The Swedish healthcare system is a decentralized tax-funded system with 21 regions responsible for the financing and provision of healthcare. Stockholm is the largest region with 2.3 million residents in 2018. Primary healthcare is generally recognized as the foundation of the healthcare system. However, the use of primary healthcare and the resources allocated to the sector are limited in comparison to healthcare systems in similar countries [19]. Low accessibility is considered as one of the main issues in the Swedish healthcare system and waiting times of six weeks are not unusual when booking a visit at a primary healthcare centre [3].

In contrast, telemedicine providers have offered almost immediate access to qualified assessments by physicians or other healthcare professionals. The high accessibility has been perceived as one of the most positive aspects of telemedicine [12]. Telemedicine consultations are part of the publicly financed healthcare system as a consequence of the Patient Act (2014:821) which was implemented in Sweden in 2015. The act entitles patients to seek outpatient care, including primary healthcare, in any region in the entire country. Following this entitlement, DTC telemedicine providers were able to reach patients from all over Sweden and starting in 2016, the use of telemedicine consultations increased rapidly. In the period 2016–2018 the majority of the telemedicine consultations consumed by the residents of Stockholm were produced by providers located in Region Jönköping, which was the first region to equate telemedicine consultations to face-to-face visits in terms of reimbursement.

### Definitions and variables

We define DTC telemedicine consultations as direct contacts between patients and healthcare personnel who are spatially separated. The contacts can take place in real time or asynchronously and should aim to correspond to a traditional primary care visit. The contacts may be in the form of a video visit, a telephone contact or an electronic chat. The DTC telemedicine consultations in this study are produced by providers targeting consumers in the entire nation with limited connection to the local healthcare system in Region Stockholm.

To explore the determinants for use of DTC telemedicine consultations, we compare it to the use of three other types of healthcare contacts. The first comparator are face-to-face physician visits which are defined as physician visits at a local primary healthcare centre. The second comparator are video consultations produced by local primary healthcare centres. During the study period, some primary healthcare centres had initialized video consultations in addition to the face-to-face consultations. We refer to these contacts as digi-physical telemedicine consultations because the same provider produces both digital telemedicine consultations and face-to-face visits depending on the need of the patient. The digi-physical telemedicine consultations are much fewer in number than the DTC telemedicine consultations (although they have increased substantially during the COVID-19 pandemic) but make an interesting comparator because of their integration in the health care system. The consultations are presumably consumed to a greater extent by patients who are already in contact with the healthcare system and are most likely initiated by the provider rather than the patient. The third type of healthcare contacts are nurse telephone consultations to the national number 1177. These consultations have been a part of the provision of healthcare services for many years and consist of assessments of the need for care, healthcare advice and guidance to the appropriate care clinic when needed. The contacts have some elements in common with DTC telemedicine; the contact is initiated by the patient and the response is immediate or within a short waiting time. However, the calls are answered by nurses instead of physicians, they do not aim to correspond to a regular healthcare contact and there is no patient fee. Table 1 gives an overview of the four types of healthcare contacts included in the study.

**Table 1:** Description of healthcare contacts categories included in the study

	**Direct-to-consumer telemedicine physician consultations**	**Face-to-face physician office visits**	**Digi-physical physician consultations**	**Nurse telephone consultations**
Provider and patient spatially separated	Yes	No	Yes	Yes
Consists of, or aims to correspond to, a traditional primary healthcare visit	Yes	Yes	Yes	No
Patient fee in 2018	SEK 250, SEK 0 for ages < 20 years and > 85 years	SEK 200, SEK 0 for ages < 18 years and > 85 years	SEK 200, SEK 0 for ages < 18 years and > 85 years	SEK 0
Produced in Stockholm	No, produced in Region Jönköping	Yes	Yes	Regional collaboration
Source	Jönköping regional healthcare database	Stockholm regional healthcare database	Stockholm regional healthcare database	Stockholm regional healthcare database

We constructed a database by linking national and regional registries from Region Jönköping (where the majority of the DTC telemedicine providers were located in 2018), Region Stockholm and Statistics Sweden. The study population initially included all residents in Region Stockholm, Sweden by December 31st, 2017 (*N* = 2.3 million).

Healthcare consultations in both digital and office settings were included in the database for the year 2018. DTC telemedicine consultations were collected from Region Jönköping regional healthcare database while all other healthcare consumption was collected from the Stockholm regional healthcare database Vårdanalysdatabasen (VAL). Both databases contain individual level data on the healthcare visits produced in respective region.

We also included individual measures of morbidity and socioeconomic variables in the database. To construct variables on presence of chronic conditions we scanned the VAL database for historic healthcare consumption for the study population during the period 2013–2017. We selected four common chronic conditions in primary healthcare: heart failure, depression, diabetes and COPD/asthma. If an individual had at least one registered diagnosis for any of these conditions during the five-year period, then the individual was categorised into that diagnosis group. Socioeconomic variables regarding education, country of birth and income were collected from Statistics Sweden. Education and country of birth were categorized into three versus four categories. Individuals under the age of 25 were given the same educational attainment as the parent with the highest education since a large proportion of this group has not finished their education. For income, we used the household disposable income weighted by the number of household members for each individual. The income variable was then ranked into ten groups where 10 represents the ten percent with the highest income and 1 the ten percent with the lowest income.

In addition, we included information of chosen primary healthcare provider. Choice of provider was observed in December 2018 and collected from the VAL database. To measure distance to provider we estimated the straight-line distance in kilometres from the residential location to the provider. Stockholm is divided into approximately 1,400 small areas with, on average, 1,600 residents. We assumed the geographical coordinates for the centroid of each small area as the residential location for the individuals living in that area. Distance was grouped into five categories (0–1 km, 1–2 km, 2–4 km, 4–10 km and 10 + km).

Moreover, we linked data on accessibility of the chosen primary healthcare for each individual. Accessibility data were collected from a large patient satisfaction survey aimed at primary care patients [20]. The survey includes five questions related to the accessibility of the primary health care centre, both in terms of accessing the facility as well as getting an appointment within a reasonable time and the staff's accessibility for the patient. The results are presented as the percentages of positive respondents to the questions and are weighted together to an index (range 0–100). We ranked all primary health care according to their results and grouped the accessibility into three categories (low, medium, high). The grouping was based on percentiles in order to make them similar in size. Index values below or equal to 78 were classified as low accessibility, index values above 78 and below or equal to 84 were classified as medium and index values above 84 were classified as high.

We excluded individuals that had missing data on income or education and those that were not registered with a primary health care provider in 2018. The final sample consists of 1 991 995 individuals (87% of the entire population in Region Stockholm) according to the distribution in Table 2.

**Table 2:** Distribution of study population, number of individuals and percent

		Number of individuals	Percent
Sex	Men	979 292	49.2
	Women	1 012 703	50.8
Age group	0–5	137 630	6.9
	6–18	319 725	16.1
	19–25	145 556	7.3
	26–45	582 611	29.3
	46–64	485 603	24.4
	65 +	320 870	16.1
Highest completed level of education	Lower secondary education	209 337	10.5
	Upper secondary education	701 776	35.2
	Post-secondary education less than 3 years	344 043	17.3
	Post-secondary education 3 years or more	736 839	37.0
Country of birth	Sweden	1 568 003	78.7
	EU28	136 105	6.8
	Outside EU28	287 887	14.5
Income group	1 (lowest income)	150 864	7.6
	2	191 279	9.6
	3	204 422	10.3
	4	209 992	10.5
	5	211 889	10.6
	6	209 971	10.5
	7	209 316	10.5
	8	205 066	10.3
	9	201 489	10.1
	10 (highest income)	197 707	9.9
Diagnoses for chronic conditions in 2013–2017	Heart failure	19 592	1.0
	Depression	150 642	7.6
	Diabetes	88 181	4.4
	COPD/asthma	164 189	8.2
Primary healthcare centre accessibility	Low	648 816	32.6
	Medium	704 136	35.4
	High	639 043	32.1
Distance to chosen primary healthcare centre	0–1 km	777 852	39.1
	1–2 km	455 066	22.8
	2–4 km	341 395	17.1
	4–10 km	245 409	12.3
	10 + km	172 273	8.7
Total		1 991 995	100

### Statistical analysis

To investigate the characteristics of DTC telemedicine users in comparison to consumers of other healthcare contacts, we applied multivariate logistic regression models. Separate models for the different types of healthcare contacts were specified. The dependent variables in the four models consisted of the binary variable of having made at least one visit during 2018 in respective healthcare category. The results are presented as odds ratios of the odds for each group in relation to the reference group. Since individuals are clustered within primary healthcare providers their characteristics are not independent. To adjust for the intra-cluster correlation, robust standard errors were computed using the empirical (“sandwich”) estimator. All analyses were conducted in SAS Enterprise Guide 7.1.

## Results

**Fig. 1:**
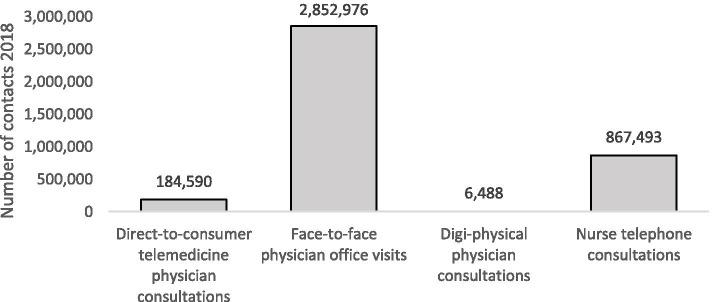
Number of contacts in the four categories included in the study in 2018

Although the number of telemedicine consultations has increased substantially since the introduction in 2016, the number of consultations was still low in comparison to physician office visits in primary care in 2018 (Fig. 1). The number of physician office visits was 15 times as high as the number of DTC telemedicine consultations and digi-physical consultations combined.

Table 3 shows the odds ratios of the odds of having made at least one contact in each of the four categories in 2018. An odds ratio and a confidence interval (CI) above one indicate a greater likelihood of having made a healthcare contact in comparison to the reference group.

**Table 3:** Odds ratio estimates of the odds of having made at least one healthcare contact in four categories in 2018 for the residents of Region Stockholm, all ages

	Direct-to-consumer telemedicine physician consultations			Face-to-face physician office visits			Digi-physical telemedicine physician consultations			Nurse telephone consultations		
	Odds ratios	95% CI		Odds ratios	95% CI		Odds ratios	95% CI		Odds ratios	95% CI	
Women vs men	1.60	1.58	1.62	1.46	1.45	1.47	1.61	1.52	1.71	1.48	1.47	1.49
Age group 0–5 vs 19–25	1.87	1.83	1.92	1.51	1.49	1.54	1.03	0.89	1.20	2.15	2.11	2.18
Age group 6–18 vs 19–25	0.74	0.72	0.76	0.92	0.91	0.93	0.74	0.65	0.84	0.70	0.69	0.71
Age group 26–45 vs 19–25	0.82	0.81	0.84	1.15	1.13	1.16	1.34	1.20	1.50	0.79	0.78	0.80
Age group 46–64 vs 19–25	0.30	0.29	0.31	1.69	1.66	1.71	1.23	1.09	1.38	0.51	0.51	0.52
Age group 65 + vs 19–25	0.07	0.06	0.07	3.81	3.76	3.87	0.51	0.44	0.59	0.66	0.65	0.67
Country of birth EU28 vs Sweden	0.63	0.61	0.65	1.00	0.99	1.01	0.58	0.50	0.66	0.76	0.75	0.77
Country of birth outside EU28 vs Sweden	0.53	0.52	0.55	1.31	1.30	1.32	0.51	0.46	0.56	0.84	0.83	0.85
Upper secondary education vs lower secondary	1.34	1.30	1.38	0.97	0.96	0.98	1.36	1.21	1.54	1.07	1.06	1.09
Post-secondary less than 3 years vs lower secondary	1.51	1.47	1.56	0.94	0.93	0.95	1.28	1.12	1.46	1.12	1.10	1.13
Post-secondary 3 years or more vs lower secondary	1.38	1.34	1.42	0.86	0.85	0.87	1.22	1.07	1.38	1.10	1.09	1.12
Income group 2 vs 1 (lowest income)	1.19	1.15	1.24	1.20	1.18	1.22	1.18	0.99	1.40	1.26	1.23	1.28
Income group 3 vs 1	1.42	1.37	1.46	1.24	1.22	1.26	1.43	1.21	1.68	1.29	1.26	1.31
Income group 4 vs 1	1.58	1.53	1.63	1.25	1.23	1.27	1.57	1.34	1.84	1.27	1.25	1.29
Income group 5 vs 1	1.72	1.67	1.78	1.21	1.20	1.23	1.55	1.32	1.81	1.26	1.24	1.28
Income group 6 vs 1	1.77	1.71	1.83	1.17	1.15	1.19	1.71	1.46	1.99	1.21	1.19	1.23
Income group 7 vs 1	1.86	1.80	1.92	1.11	1.10	1.13	1.56	1.34	1.83	1.17	1.15	1.19
Income group 8 vs 1	1.99	1.93	2.06	1.07	1.05	1.09	1.71	1.46	2.01	1.14	1.12	1.16
Income group 9 vs 1	2.11	2.05	2.18	1.01	0.99	1.02	1.61	1.37	1.89	1.11	1.09	1.13
Income group 10 (highest income) vs 1	2.46	2.38	2.54	0.89	0.87	0.90	2.02	1.73	2.37	1.09	1.07	1.11
Heart failure vs no heart failure	0.49	0.40	0.59	1.45	1.40	1.51	0.56	0.33	0.93	1.81	1.75	1.87
Depression vs no depression	1.47	1.44	1.50	2.17	2.14	2.19	1.99	1.83	2.15	1.56	1.54	1.58
Diabetes vs no diabetes	0.76	0.72	0.80	2.75	2.70	2.81	0.99	0.84	1.17	1.21	1.19	1.23
COPD/asthma vs no COPD/asthma	1.37	1.34	1.40	1.79	1.76	1.81	1.59	1.46	1.74	1.38	1.37	1.40
Accessibility medium vs low	0.97	0.96	0.98	1.20	1.20	1.21	0.83	0.78	0.88	0.97	0.96	0.98
Accessibility high vs low	0.88	0.87	0.90	1.37	1.36	1.38	0.35	0.32	0.38	0.91	0.90	0.92
Distance 1–2 km vs 0–1 km	1.05	1.04	1.07	0.97	0.97	0.98	1.38	1.27	1.50	1.02	1.02	1.03
Distance 2–4 km vs 0–1 km	1.11	1.09	1.13	0.95	0.94	0.96	2.35	2.17	2.55	1.03	1.02	1.04
Distance 4–10 km vs 0–1 km	1.09	1.07	1.11	0.87	0.87	0.88	2.32	2.13	2.53	1.00	0.99	1.01
Distance 10 + km vs 0–1 km	1.02	1.00	1.05	0.74	0.73	0.75	2.34	2.11	2.58	0.93	0.92	0.94

In Supplementary Table 1, the absolute numbers and percentages of users in each subgroup are presented

According to the model, patient factors that increased the likelihood of having made at least one DTC telemedicine consultation in 2018 were: being a woman, being of younger age, being born in Sweden, having a higher educational attainment and income and having had a history of depression or COPD/asthma.

Factors that increased the likelihood of having made a physician office visit differed to a great extent from the DTC telemedicine consultations. A history of heart failure and diabetes, being born outside of EU28, having a lower educational attainment, a lower level of income, and being in the age group 65 + were factors that increased the likelihood of making a physician office visits but decreased the likelihood of making a DTC telemedicine consultation.

The pattern for use of digi-physical consultations, integrated in the healthcare system, was similar to the use of DTC telemedicine consultations. The age pattern differed to some extent, but the associations related to education and country of birth were similar. The association between income and consumption of digi-physical visits was similar but not as pronounced as for the DTC option.

For telephone nurse consultations, the association between utilization and age, country of birth and education was similar to the DTC telemedicine consultations. However, the association between utilization and income differed significantly. In contrast to the results for use of DTC telemedicine, increased income was associated with a lower probability of making a call to 1177. The only exception was income group 1 which had the lowest utilization of all income groups.

Regarding the factors related to the chosen regular primary healthcare provider, a greater distance to chosen provider seemed to increase the likelihood of making a DTC telemedicine consultation and a digi-physical visit. In contrast, greater distance to chosen provider decreased the likelihood of having made a physician office visit. For accessibility, a low accessibility of the chosen provider increased the likelihood of making a DTC telemedicine consultation, a digi-physical consultation and a telephone nurse consultation, while a low accessibility decreased the likelihood of making a physician office visit.

The associations between the explanatory variables were assessed using the Cramer’s V coefficient. The strongest association, between diabetes and age group, generated a Cramer’s V coefficient of 0.25. All other associations were below 0.19. To explore the impact of the variables age group and income, which had the strongest associations with other variables, we stratified the analysis for different age groups and estimated the main model without the income variable. These estimations generated results in the same direction as the main analysis (results shown on request).

### Sensitivity analysis

For a sensitivity analysis, we excluded individuals under the age of 18 from the study population since children can have a different pattern of consumption than adults. We also performed separate analyses for the two largest DTC telemedicine actors to see if there were any differences in determinants for consultations provided by the different companies. The analyses, presented in the Supplementary materials (Tables 2 and 3), showed no notable differences from the main analysis.

## Discussion

The aim of this study was to explore the determinants for use of DTC telemedicine consultations in a setting where telemedicine is included in the publicly funded healthcare system. Our results suggest that the use of DTC telemedicine is determined by factors that are generally not associated with greater healthcare need and they contrast to the determinants for use of face-to-face visits in primary healthcare.

The utilization pattern of the DTC telemedicine consultations raises some questions about the equity implications of the introduction of digital healthcare resources. However, in order to evaluate the impact, the whole healthcare spectrum needs to be taken into account. There is some evidence that the unit cost of a telemedicine consultation is less than the unit cost of a face-to-face visit from the perspective of the healthcare financier [21–23]. Adding the assumption that telemedicine and face-to-face consultations are perfect substitutes, we identify a possibility that a transfer from face-to-face visits to telemedicine consultations for patients with assumed lower needs could lead to increased efficiency in the system. Increased efficiency could free resources that could be spent on individuals with a higher healthcare need.

On the other hand, telemedicine consultations may also trigger additional visits and be associated with increased costs and workload in primary care [15, 24]. If digital visits do not substitute face-to-face visits but correspond to a previously unmet demand, then concerns should be raised about the distributional effects of the introduction of digital healthcare. Further research on whether, and to what extent, telemedicine consultations substitute face-to-face visits in primary healthcare is needed in order to determine the equity consequences of the distribution of digital healthcare utilization. Findings from a Swedish study indicate that users of DTC telemedicine consume more healthcare than the population at large [17]. Given that accessibility in Swedish primary healthcare is limited and the level of accessibility of the DTC telemedicine providers is high, there is a probability that DTC telemedicine consultations to a large extent meet previously unmet demand. In this case, health care resources are potentially transferred from those with higher needs to groups with lower needs. However, the optimal level of healthcare consumption, in terms of vertical equity, for different groups is neither known nor defined. It could be argued that the groups that increase their primary healthcare consumption due to DTC telemedicine were previously underserved in primary healthcare.

The finding that individuals with a previous history of COPD/asthma and depression use DTC telemedicine and digi-physical consultations to a larger extent than those without these diagnoses could indicate that there is a potential for telemedicine as a part of the provision of care for these patient groups. A survey study targeting Norwegian general practitioners during the COVID-19 lockdown gives support for the potential of telemedicine for treating patients with mental illnesses [25].

Even though Sweden has a population with a high level of digital competence [26], the technology does not seem to have reached a large part of the elderly. Limited familiarity and confidence in digital technology is most likely a part of the explanation. The internet coverage in Swedish homes is 94 percent. However, about 10 percent of the population do not connect on a daily basis and the strongest determinant for not connecting is age. Two thirds of those who do not connect daily are 65 years of age or older. E-identification, which is often a prerequisite for accessing digital healthcare services, is also used to a lesser extent by the elderly. The most common form of e-identification is used by 85 percent of the population 16–85 years of age but only by 47 percent of individuals aged 75–85 [27].

Another explanation to the low use of telemedicine among elderly could be the morbidity pattern. For patients with complex multi-morbidity, continuity of care is most likely considered an important aspect. DTC telemedicine consultations, with limited connection to the local health system, could therefore be perceived as less useful for this patient group. However, digi-physical online consultations with a regular healthcare provider, as a complement to face-to-face consultations, could provide benefits for elderly patients as well. In a qualitative study with interviews of Swedish physicians providing telemedicine consultations, the participants raised concerns about the risk of generating “unnecessary” healthcare. In line with our results, the participants noted that telemedicine consultations primarily reached young and relatively healthy individuals which could possibly be at the expense of the elderly population with a higher burden of disease [5]. Policy makers should consider measures to increase access for the elderly. With an increased digitization in healthcare, it is important to make sure that there are groups in the population that are not left underserved. The accessibility for this group might be improved by providing technical support to those with limited familiarity with new technologies [28].

Another group that has a particular low telemedicine consumption in comparison to their use of traditional primary care are individuals born outside of Sweden. We suggest that policy makers investigate why individuals born outside of Sweden are underrepresented in telemedicine use and examine measures to promote use in this group in order to ensure equal access to care.

Regarding the impact of distance on use of telemedicine, previous studies have found that use of telemedicine is higher in urban populations [11, 14, 16] and that telemedicine patients had a longer travel distance to their healthcare provider in comparison to those who made a face-to-face visit [10]. Our results suggest that, within the urban region of Stockholm, a greater distance to chosen provider increases the likelihood of using telemedicine, which adds to the understanding of the consumption pattern.

Telemedicine in healthcare is a new practice and there is still lack of knowledge regarding the determinants for telemedicine utilization. The strength of the study is the unique data base with linked individual level information on healthcare utilization and individual characteristics for more than 2 million individuals. The study also has a few limitations. The setting includes a digitally competent population and national coverage of the telemedicine provision, therefore the generalizability to other settings with less digital literacy is limited. Still, the development of the telemedicine market in Swedish health care provides lessons and experience of introducing the technology in a publicly funded health system. Consequently, the findings of the differences in uptake of the technology in the population can be expected to be present in other settings with less digital literacy as well. Another limitation is the number of digi-physical visits which are very few in the study period. The results for this group should therefore be interpreted with caution. A third limitation is the timeframe of the study which comes before the COVID-19 pandemic. The COVID-19 pandemic has drastically increased the consumption of virtual care and future studies covering the pandemic period are needed to examine if our findings of differences between groups have been emphasized or reduced during this period of rapid change.

## Conclusions

There are significant differences between the levels of use of DTC telemedicine in different subgroups of the population in Stockholm, Sweden. Younger age, higher income, higher education and being born in Sweden are determinants associated with use of DTC telemedicine. The findings are consistent across different DTC providers and across other digital healthcare contacts. However, the use of DTC telemedicine is associated with factors contrasting to the determinants for use of face-to-face visits. In the future development of the digital services, policy makers should consider measures to promote access to the groups in the population with a particularly low level of consumption in order to ensure that all groups have access to the health service that best suits their needs. Further research on the impact of telemedicine on the healthcare consumption as a whole is needed in order to determine the equity consequences of the utilization pattern.

## Supplementary Information

**Additional file 1: Supplementary Table 1**. Number of individuals in each subgroup of the population who made at least one healthcare contact in four categories in 2018. **Supplementary Table 2**. Odds ratio estimates of the odds of having made at least one direct-to-consumer telemedicine physician consultation in 2018 for residents of Region Stockholm above 18 years of age. **Supplementary Table 3**. Odds ratio estimates of the odds of having made at least one direct-to-consumer telemedicine physician consultation in 2018 for residents of Region Stockholm, by provider

## Data Availability

The data were obtained from Statistics Sweden, Region Stockholm and Region Jönköping. Due to legal restrictions, we are prevented from making the data publicly available or otherwise sharing individual level data. For access to data for research purposes, please contact the owners of the data.

## Abbreviations

DTC: Direct-to-consumer
CI: Confidence intervals
