# Supplementary material for: Determinants for use of direct-to-consumer telemedicine consultations in primary healthcare—a registry based total population study from Stockholm, Sweden
Source: BMC Fam Pract. 2021 Jun 26;22:133. doi: 10.1186/s12875-021-01481-1 (PMC8233176; doi:10.1186/s12875-021-01481-1)
Supplement: Supplementary file 1 — Additional file 1: Supplementary Table 1. Number of individuals in each subgroup of the population who made at least one healthcare contact in four categories in 2018. Supplementary Table 2. Odds ratio estimates of the odds of having made at least one direct-to-consumer telemedicine physician consultation in 2018 for residents of Region Stockholm above 18 years of age. Supplementary Table 3. Odds ratio estimates of the odds of having made at least one direct-to-consumer telemedicine physician consultation in 2018 for residents of Region Stockholm, by provider [file 12875_2021_1481_MOESM1_ESM.docx]

Supplementary material

Determinants for use of direct-to-consumer telemedicine consultations in primary healthcare

A registry based total population study from Stockholm, Sweden

Cecilia Dahlgren^1,2^, Margareta Dackehag^3^, Per Wändell^4^, Clas Rehnberg^1^

1. Karolinska Institutet, Department of Learning, Informatics, Management and Ethics, Stockholm, SE
2. Region Stockholm, Center for Health Economics, Informatics and Healthcare Research, Stockholm, SE
3. Lund University, Department of Economics, Lund, SE
4. Karolinska Institutet, Department of Neurobiology, Care Sciences and Society, Division of Family Medicine and Primary Care, Stockholm, SE

Corresponding Author: Cecilia Dahlgren

Address: Karolinska Institutet

Tomtebodavägen 18 A, plan 4 LIME

171 77 Stockholm

E-mail: cecilia.dahlgren@ki.se

Phone number: +46 702 36 32 74

**Supplementary table 1. Number of individuals in each subgroup of the population who made at least one healthcare contact in four categories in 2018**

|  |  | Direct-to-consumer telemedicine physician consultations | | Face-to-face physician office visits | | Digi-physical telemedicine physician consultations | | Nurse telephone consultations | |
| --- | --- | --- | --- | --- | --- | --- | --- | --- | --- |
|  |  | Number of individuals | % | Number of individuals | % | Number of individuals | % | Number of individuals | % |
| Sex | Men | 49 769 | 5.1 | 484 292 | 49.5 | 1 844 | 0.2 | 187 220 | 19.1 |
|  | Women | 76 198 | 7.5 | 602 486 | 59.5 | 3 090 | 0.3 | 262 177 | 25.9 |
| Age group | 0-5 | 23 046 | 16.7 | 72 843 | 52.9 | 325 | 0.2 | 61 819 | 44.9 |
|  | 6-18 | 24 487 | 7.7 | 131 534 | 41.1 | 564 | 0.2 | 66 933 | 20.9 |
|  | 19-25 | 14 477 | 9.9 | 64 470 | 44.3 | 388 | 0.3 | 40 512 | 27.8 |
|  | 26-45 | 45 962 | 7.9 | 283 483 | 48.7 | 1 859 | 0.3 | 133 261 | 22.9 |
|  | 46-64 | 15 836 | 3.3 | 284 019 | 58.5 | 1 442 | 0.3 | 78 586 | 16.2 |
|  | 65+ | 2 159 | 0.7 | 250 429 | 78.0 | 356 | 0.1 | 68 286 | 21.3 |
| Highest completed level of education | Lower secondary | 5 336 | 2.5 | 133 272 | 63.7 | 323 | 0.2 | 41 368 | 19.8 |
|  | Upper secondary | 38 487 | 5.5 | 399 397 | 56.9 | 1 919 | 0.3 | 153 807 | 21.9 |
|  | Post-secondary less than 3 years | 24 713 | 7.2 | 184 977 | 53.8 | 883 | 0.3 | 79 827 | 23.2 |
|  | Post-secondary 3 years or more | 57 431 | 7.8 | 369 132 | 50.1 | 1 809 | 0.2 | 174 395 | 23.7 |
| Country of birth | Sweden | 112 658 | 7.2 | 836 574 | 53.4 | 4 292 | 0.3 | 370 997 | 23.7 |
|  | EU28 | 4 185 | 3.1 | 80 429 | 59.1 | 207 | 0.2 | 23 775 | 17.5 |
|  | Outside EU28 | 9 124 | 3.2 | 169 775 | 59.0 | 435 | 0.2 | 54 625 | 19.0 |
| Income group | 1 (lowest income) | 6 097 | 4.0 | 77 198 | 51.2 | 225 | 0.1 | 30 808 | 20.4 |
|  | 2 | 7 978 | 4.2 | 116 459 | 60.9 | 324 | 0.2 | 46 957 | 24.5 |
|  | 3 | 10 884 | 5.3 | 121 857 | 59.6 | 438 | 0.2 | 51 654 | 25.3 |
|  | 4 | 13 083 | 6.2 | 121 345 | 57.8 | 526 | 0.3 | 52 168 | 24.8 |
|  | 5 | 14 676 | 6.9 | 117 813 | 55.6 | 551 | 0.3 | 51 452 | 24.3 |
|  | 6 | 14 747 | 7.0 | 113 401 | 54.0 | 613 | 0.3 | 48 480 | 23.1 |
|  | 7 | 14 807 | 7.1 | 110 572 | 52.8 | 551 | 0.3 | 46 067 | 22.0 |
|  | 8 | 14 532 | 7.1 | 107 106 | 52.2 | 579 | 0.3 | 43 022 | 21.0 |
|  | 9 | 14 001 | 6.9 | 103 682 | 51.5 | 517 | 0.3 | 40 051 | 19.9 |
|  | 10 (highest income) | 15 162 | 7.7 | 97 345 | 49.2 | 610 | 0.3 | 38 738 | 19.6 |
| Diagnoses for chronic conditions in 2013-2017 | Heart failure | 103 | 0.5 | 16 218 | 82.8 | 15 | 0.1 | 6 238 | 31.8 |
|  | No heart failure | 125 864 | 6.4 | 1 070 560 | 54.3 | 4 919 | 0.2 | 443 159 | 22.5 |
|  | Depression | 11 676 | 7.8 | 109 429 | 72.6 | 776 | 0.5 | 44 984 | 29.9 |
|  | No depression | 114 291 | 6.2 | 977 349 | 53.1 | 4 158 | 0.2 | 404 413 | 22.0 |
|  | Diabetes | 1 407 | 1.6 | 74 123 | 84.1 | 151 | 0.2 | 19 702 | 22.3 |
|  | No diabetes | 124 560 | 6.5 | 1 012 655 | 53.2 | 4 783 | 0.3 | 429 695 | 22.6 |
|  | COPD/asthma | 13 190 | 8.0 | 113 102 | 68.9 | 597 | 0.4 | 48 427 | 29.5 |
|  | No COPD/asthma | 112 777 | 6.2 | 973 676 | 53.3 | 4 337 | 0.2 | 400 970 | 21.9 |
| Primary healthcare centre accessibility | Low | 41 747 | 6.4 | 333 301 | 51.4 | 2 067 | 0.3 | 152 143 | 23.4 |
|  | Medium | 44 762 | 6.4 | 387 337 | 55.0 | 2 052 | 0.3 | 159 038 | 22.6 |
|  | High | 39 458 | 6.2 | 366 140 | 57.3 | 815 | 0.1 | 138 216 | 21.6 |
| Distance to chosen primary healthcare centre | 0-1 km | 44 973 | 5.8 | 442 134 | 56.8 | 1 162 | 0.1 | 176 433 | 22.7 |
|  | 1-2 km | 29 831 | 6.6 | 250 164 | 55.0 | 984 | 0.2 | 104 836 | 23.0 |
|  | 2-4 km | 24 294 | 7.1 | 183 248 | 53.7 | 1 282 | 0.4 | 78 443 | 23.0 |
|  | 4-10 km | 16 848 | 6.9 | 127 241 | 51.8 | 908 | 0.4 | 54 653 | 22.3 |
|  | 10+ km | 10 021 | 5.8 | 83 991 | 48.8 | 598 | 0.3 | 35 032 | 20.3 |
| Total |  | 125 967 | 6.3 | 1 086 778 | 54.6 | 4 934 | 0.2 | 449 397 | 22.6 |

**Supplementary table 2. Odds ratio estimates of the odds of having made at least one direct-to-consumer telemedicine physician consultation in 2018 for residents of Region Stockholm above 18 years of age**

|  | Direct-to-consumer telemedicine physician consultations | | | | |
| --- | --- | --- | --- | --- | --- |
|  | | | Odds Ratios | 95% Confidence Interval | |
| Women vs Men | | 1.89 | | 1.86 | 1.91 |
| Age group 26-45 vs 19-25 | | 0.82 | | 0.81 | 0.84 |
| Age group 46-64 vs 19-25 | | 0.30 | | 0.29 | 0.31 |
| Age group 65+ vs 19-25 | | 0.07 | | 0.06 | 0.07 |
| Country of birth EU28 vs Sweden | | 0.62 | | 0.60 | 0.64 |
| Country of birth outside EU28 vs Sweden | | 0.52 | | 0.51 | 0.53 |
| Upper secondary vs lower secondary | | 1.28 | | 1.24 | 1.32 |
| Post-secondary less than 3 years vs lower secondary | | 1.46 | | 1.41 | 1.51 |
| Post-secondary 3 years or more vs lower secondary | | 1.34 | | 1.30 | 1.39 |
| Income group 2 vs 1 (lowest income) | | 1.12 | | 1.07 | 1.17 |
| Income group 3 vs 1 | | 1.31 | | 1.25 | 1.36 |
| Income group 4 vs 1 | | 1.43 | | 1.38 | 1.49 |
| Income group 5 vs 1 | | 1.56 | | 1.50 | 1.62 |
| Income group 6 vs 1 | | 1.61 | | 1.55 | 1.68 |
| Income group 7 vs 1 | | 1.68 | | 1.62 | 1.75 |
| Income group 8 vs 1 | | 1.81 | | 1.74 | 1.88 |
| Income group 9 vs 1 | | 1.92 | | 1.84 | 2.00 |
| Income group 10 (highest income) vs 1 | | 2.29 | | 2.20 | 2.38 |
| Heart failure vs no heart failure | | 0.48 | | 0.40 | 0.59 |
| Depression vs no depression | | 1.42 | | 1.39 | 1.45 |
| Diabetes vs no diabetes | | 0.76 | | 0.72 | 0.81 |
| COPD/asthma vs no COPD/asthma | | 1.36 | | 1.32 | 1.40 |
| Accessibility medium vs low | | 0.99 | | 0.97 | 1.01 |
| Accessibility high vs low | | 0.87 | | 0.85 | 0.88 |
| Distance 1-2 km vs 0-1 km | | 1.02 | | 1.00 | 1.04 |
| Distance 2-4 km vs 0-1 km | | 1.06 | | 1.04 | 1.08 |
| Distance 4-10 km vs 0-1 km | | 1.08 | | 1.05 | 1.10 |
| Distance 10+ km vs 0-1 km | | 1.00 | | 0.98 | 1.03 |

**Supplementary table 3. Odds ratio estimates of the odds of having made at least one direct-to-consumer telemedicine physician consultation in 2018 for residents of Region Stockholm, by provider**

|  | Provider A: Direct-to-consumer telemedicine physician consultations | | | Provider B: Direct-to-consumer telemedicine physician consultations | | |
| --- | --- | --- | --- | --- | --- | --- |
|  | Odds Ratios | 95% CI | | Odds Ratios | 95% CI | |
| Women vs Men | 1.54 | 1.52 | 1.56 | 1.79 | 1.75 | 1.84 |
| Age group 0-5 vs 19-25 | 2.19 | 2.13 | 2.24 | 0.98 | 0.93 | 1.03 |
| Age group 6-18 vs 19-25 | 0.80 | 0.78 | 0.82 | 0.57 | 0.54 | 0.59 |
| Age group 26-45 vs 19-25 | 0.83 | 0.81 | 0.85 | 0.81 | 0.78 | 0.84 |
| Age group 46-64 vs 19-25 | 0.27 | 0.26 | 0.28 | 0.39 | 0.37 | 0.41 |
| Age group 65+ vs 19-25 | 0.06 | 0.05 | 0.06 | 0.09 | 0.08 | 0.10 |
| Country of birth EU28 vs Sweden | 0.60 | 0.58 | 0.63 | 0.70 | 0.66 | 0.75 |
| Country of birth outside EU28 vs Sweden | 0.53 | 0.51 | 0.54 | 0.56 | 0.53 | 0.58 |
| Upper secondary education vs lower secondary | 1.39 | 1.34 | 1.44 | 1.24 | 1.18 | 1.32 |
| Post-secondary less than 3 years vs lower secondary | 1.63 | 1.58 | 1.70 | 1.21 | 1.14 | 1.28 |
| Post-secondary 3 years or more vs lower secondary | 1.50 | 1.45 | 1.55 | 1.03 | 0.98 | 1.10 |
| Income group 2 vs 1 (lowest income) | 1.19 | 1.15 | 1.24 | 1.23 | 1.14 | 1.32 |
| Income group 3 vs 1 | 1.42 | 1.37 | 1.47 | 1.37 | 1.28 | 1.47 |
| Income group 4 vs 1 | 1.59 | 1.54 | 1.65 | 1.51 | 1.41 | 1.61 |
| Income group 5 vs 1 | 1.76 | 1.69 | 1.82 | 1.56 | 1.46 | 1.67 |
| Income group 6 vs 1 | 1.81 | 1.74 | 1.87 | 1.61 | 1.51 | 1.72 |
| Income group 7 vs 1 | 1.90 | 1.83 | 1.97 | 1.65 | 1.55 | 1.77 |
| Income group 8 vs 1 | 2.07 | 1.99 | 2.14 | 1.68 | 1.57 | 1.80 |
| Income group 9 vs 1 | 2.22 | 2.14 | 2.30 | 1.75 | 1.63 | 1.87 |
| Income group 10 (highest income) vs 1 | 2.60 | 2.51 | 2.70 | 1.95 | 1.82 | 2.09 |
| Heart failure vs no heart failure | 0.56 | 0.44 | 0.70 | 0.34 | 0.22 | 0.53 |
| Depression vs no depression | 1.43 | 1.39 | 1.46 | 1.46 | 1.40 | 1.52 |
| Diabetes vs no diabetes | 0.73 | 0.69 | 0.78 | 0.77 | 0.70 | 0.86 |
| COPD/asthma vs no COPD/asthma | 1.36 | 1.33 | 1.39 | 1.31 | 1.26 | 1.37 |
| Accessibility medium vs low | 0.97 | 0.96 | 0.99 | 0.98 | 0.95 | 1.00 |
| Accessibility high vs low | 0.92 | 0.91 | 0.94 | 0.85 | 0.82 | 0.87 |
| Distance 1-2 km vs 0-1 km | 1.06 | 1.05 | 1.08 | 1.04 | 1.00 | 1.08 |
| Distance 2-4 km vs 0-1 km | 1.13 | 1.11 | 1.15 | 1.09 | 1.05 | 1.13 |
| Distance 4-10 km vs 0-1 km | 1.08 | 1.06 | 1.10 | 1.15 | 1.11 | 1.20 |
| Distance 10+ km vs 0-1 km | 0.99 | 0.96 | 1.01 | 1.17 | 1.12 | 1.23 |
